# Outcomes of two different treatment modalities in mild to moderate keratoconus

**DOI:** 10.1186/s12886-023-03040-x

**Published:** 2023-07-17

**Authors:** Ahmed M Khalafallah, Mohamed F. Abdelkader, Ahmed M Sabry, Yahia M Khairat, Ahmed A Abdelghany

**Affiliations:** Ophthalmology Department, Faculty of Medicine, Minia University, Minia, Egypt

**Keywords:** Keratoconus, Intracorneal ring segment, Toric ICL, Corneal collagen crosslinking

## Abstract

**Purpose:**

To describe visual and refractive outcomes of intrastromal corneal ring segments (ICRS) and toric implantable collamer lenses (TICL) implantation in cases of mild and moderate keratoconus.

**Methods:**

A prospective descriptive interventional case series. 40 eyes were allocated into two groups. First group (20 eyes) was treated with corneal collagen crosslinking (CXL) 1 month after ICRS implantation and the second group was treated using TICL after 1 year of CXL.

**Results:**

Both groups showed statistically significant improvement in spherical equivalent, cylindrical refraction, uncorrected visual acuity (UCVA) and best corrected visual acuity (BCVA) over the follow-up period.

**Conclusion:**

Both ICRS and TICL are effective in treatment of mid and moderate keratoconus with more predictable visual results with TICL.

## Introduction

Keratoconus is a progressive non-inflammatory corneal ectatic disease which is characterized by bilateral asymmetric progressive diminution of vision with a prevalence reaching more than 26 per 10,000 [1].

Spectacles and hard contact lenses are appropriate treatment options in mild to moderate stages [2]. However, keratoplasty may be required in advanced stages, with the well-known disadvantages such as the high cost, lifelong follow-up visits, the graft and suture-related complications [3, 4].

Other surgical options were developed for keratoconus cases to correct its related visual disturbances. Some of these options are one or combination of corneal cross-linking, implantation of intrastromal corneal ring segment, topography-guided photorefractive keratectomy, and toric phakic intraocular lens implantation [5, 6].

In corneal collagen crosslinking (CXL); weak stromal fibers are photopolymerized to increase the collagen rigidity using ultraviolet rays (UV-A) and riboflavin. The acquired rigidity makes CXL is the most effective among those previously mentioned options to slow progression of the keratoconus or may arrest it to avoid the need for keratoplasty [5].

Intrastromal corneal ring segments (ICRSs) are devices made of polymethylmethacrylate that vary by arc length and optical zone for insertion and act like tissue add to the mid-periphery of the cornea and so, corneal ring segments regularize and flatten the cornea and thus alter the corneal refractive power to improve vision. For at least 20 years, many studies for corneal segments prove ultimate success in treatment of keratoconus with appropriate corneal parameters in most studies [7, 8].

The TICL (toric implantable collamer lens) insertion is used off-label for the treatment of non-progressive keratoconus and have myopic correction range reaching to -20 D (− 3.0 to − 20.0 D) and astigmatic correction range reaching up to 6 D. Stable refraction and anterior chamber depth of at least 3 mm is mandatory for its implantation. TICL has high successful results in the correction power with high uncorrected and best corrected visual acuity [9, 10].

## Patients and methods

This study was a prospective descriptive interventional case series. It was conducted in Minia university hospital and Roaa Laser Vision Correction Center. The study was approved by the ethical committee of Minia University, and conducted under the umbrella of the Helsinki by laws; all patients signed informed consent after full explanation of the nature of the condition and the methods of management.

Forty eyes were allocated into two groups: one of them included 20 eyes were treated by CXL (epithelium off) 1 month after Femtosecond laser assisted ICRS (Kera-rings) while the other included 20 eyes were treated by phakic TICL implantation 1 year after CXL (epithelium off).

Inclusion criteria for all these cases were keratoconus grade I to III according to the Amsler-Krumeich classification and age ranging from 21 to 35 years, TICL cases were with stable cycloplegic refraction for at least 6 months after CXLand ICRS cases were with lower cone in pentacam and irregular astigmatism with best spectacle corrected visual acuity BSCVA less than 0.5 (snellen).

All patients were subjected for history taking, slit lamp examination, cycloplegic refraction, uncorrected visual acuity (UCVA) and best corrected visual acuity (BCVA) by the Snellen chart, based on the logMAR scoring system, fundus examination, intraocular pressure (IOP) and pentacam examination: pre-operative before each surgical procedure and 3,6 months and 1 year post-operative after the second surgical procedure. To calculate safety and efficacy of both procedures, safety = BSCVA post-operative (1 year) / BSCVA preoperative and efficacy = UCVA post-operative (1 year) / BSCVA preoperative.

Cases with acute hydrops or grade IV keratoconus, keratometric readings greater than 65 Dioptre, corneal thickness less than 400 μm at the thinnest corneal point, and at least 450 μm at the incision site for ICRS group, history of herpetic keratitis, autoimmune or systemic connective tissue disease, corneal dystrophies or any corneal opacities and any ocular diseases or surgeries were excluded from the study.

Follow up visits were at: day 1 ,1 week, then monthly up to 6 month and at 1 year.

### Surgical steps

#### ICRS

After segment(s) choice according to the manufacturer nomogram and under complete aseptic conditions using topical anesthesia (Benoxinate hydrochloride 0.4% (Benox, Eipico) 3 times 90 s intervals started 10 min before the procedure), the surgical procedure was performed as follows:

Tunnel formation for Kera-Rings:

The creation of the intrastromal tunnel for KeraRing (KeraRing; Mediphacos, Belo Horizonte, Brazil) implantation was performed by means of the 150 kHz femtosecond technology (IntraLase, Abbott, California, USA) in all cases. The device was programmed with the laser software for an outer diameter of 5.9 mm and inner diameter of 5 mm. a tunnel of 80% of the thickness of the thinnest location in depth was created.

Kera-Ring implantation:

After the tunnel formation, a space was formed by spatula passing through the corneal incision where the Kera-Ring was introduced using a modified Macpherson forceps.

After Kera-Ring implantation, topical moxifloxacin (vigamox, Alcon co.) was applied. Then soft contact lens was applied.

#### CXL

After sterilization and under topical anesthesia, mechanical debridement of corneal epithelium over the central 9–10 mm (after application of diluted alcohol 20% for 35 s) was done using a blunt instrument. 0.1% Riboflavin in 20% hydroxypropyl methyl cellulose solution (vibex rapid, Avedro inc, USA) was instilled topically every 2 min for 10 min. The cornea was exposed to UVA light of 365 nm at an irradiance of 30 mW/cm^2^ for 8 min of pulsed light where every 1 s of UVA irradiation was alternated with a 1 s pause (Avedro, Avedro inc, Waltham MA, USA). Riboflavin instillation was continued every 2 min during exposure to UVA light. Antibiotic drops were administered and a soft contact lens was placed till complete re-epithelialization. Artificial tears and corticosteroid drops were used for 1 month and corticosteroid was tapered over this month.

#### TORIC ICL

Using a caliper, the toric ICL (TICL; Visian Toric ICL™; STAAR Surgical AG, Nidau, Switzerland) size was determined based on the horizontal white-to-white distance.

Marking the axis at the cornea and after pupillary dilation and under local anesthesia, a small clear temporal corneal incision and 2 paracenteses were created. The toric ICL was injected using a specific injector cartridge and unfolded slowly after viscoelastic injection. A modified ICL manipulator was used for TICL positioning under the iris and correcting its axis. After viscoelastic removal, an intracameral miotic was injected. No sutures were required at the end of the surgery.

After surgery, topical moxifloxacin (vigamox, Alcon co.) was applied. Antibiotic drops and corticosteroid drops were administered for 4 weeks with tapering of steroid 2 weeks before stoppage.

### Statistical analysis

The analysis of the data was carried out using the IBM SPSS 20.0 statistical package software (IBM; Armonk, New York, USA). Normality of the data was tested using the Shapiro-Wilk or Kolmogorov-Smirnov tests. Comparison between unrelated variables was conducted with Student’s t-test or Mann–Whitney U test, as appropriate. The chi square test was used for comparison between categorical variables. Repeated measure ANOVA followed by Bonferroni post hoc test for parametric data or Friedman test followed by Dunn’s multiple comparison tests for non-parametric were used to compare dependent data.

## Results

This study was conducted on 40 eyes of 28 patients 12 bilateral and 16 unilateral allocated evenly into two groups (ICRS group and TICL group) each of them with 20 eyes (6 bilateral and 8 unilateral) with a mean age of 27.9 for ICRS group ranging from 21 to 35 years and mean age of 26.8 for TICL group ranging from 23 to 35 years. The patients included in the study were 14 males (6 in ICRS group and 8 in TICL group) and 14 females (8 in ICRS group and 6 in TICL group). Success in ICRS implantation in keratoconic corneas was defined as decrease in mean keratometric reading more than 1 D, decrease in spherical equivalent more than 1D, increase in UCVA 1 line or more and increase in BCVA 1 line or more and which happened in all enrolled cases in this study.

Tables 1 and 2 show mean ± Standard Deviation (SD) and median of preoperative and postoperative (1 month, 3 months, 6 months and 1 year) changes in spherical equivalent, cylindrical refraction, uncorrected visual acuity (UCVA) and best spectacle corrected visual acuity (BSCVA) respectively. And as shown there are statistically significant differences in both groups between preoperative and 12 months post-operative data regarding spherical equivalent (The mean spherical equivalent improved from − 8.2 D to -5.02D in ICRS group and in TICL group improved from − 7.83D to -0.61D within 1 year), cylindrical refraction (The mean) improved from − 5.64 D to -3.24D in ICRS group and in TICL group improved from − 3.66 D to -0.51D within the same period) and UCVA (The mean UCVA (log MAR) improved from 1.15 to 0.9 in ICRS group and in TICL group improved from 1.19 to 0.31 within 1 year), while regarding BCVA (log MAR) in the postoperative final results (The mean improved from 0.57 to 0.33 in ICRS group and in TICL group improved from 0.33 to 0.27). All changes show statistically significant improvements.

**Table 1:** Results of spherical equivalent and cylindrical refraction in both groups

	Spherical equivalent		Cylindrical refraction	
	ICRS	TICL	ICRS	TICL
	N = 20	N = 20	N = 20	N = 20
**Baseline** Mean ± SD Median (Range) 95% CI	-8.2 ± 4.1 -7 (-16.25 to -3) -6.75 to – 10.12	-7.83 ± 6.16 –7.63 (-1.75to -17.75) -4.94 to -10.71	-5.64 ± 2.35 -6 (-2 to -10.5) -4.54 to -6.74	-3.66 ± 1.59 –3.5 (-1 to -6) -2.92 to -4.41
**1 m post-op** Mean ± SD Median (Range) 95% CI	# -5.35 ± 3.65 –4.63 (-0.75 to -14) -3.64 to -7.06	# -0.83 ± 0.72 (0.75 to -1.75) -0.49 to -1.16	# -3.42 ± 1.75 -3 (-0.75 to -8) -2.61 to -4.24	# -0.73 ± 0.72 -1 (-1.5 to 1) -0.39 to – 1.06
**3 m post-op** Mean ± SD Median (Range) 95% CI	# -5.6 ± 3.83 –4.63 (-0.25 to -15.25) -3.81 to -7.39	# -0.79 ± 0.61 -1 (-1.5 to 0.75) -0.5 to -1.07	# -3.67 ± 1.95 –3.13 (-0.75 to -9) -2.76 to -4.59	# -0.62 ± 0.55 –0.75 (-1.25 to 1) -0.37 to -0.88
**6 m post-op** Mean ± SD Median (Range) 95% CI	# -5.04 ± 3.67 -4 (-13 to 0.5) -3.32 to -6.76	# -0.46 ± 0.49 –0.5 (-1 to 0.5) -0.23 to -0.69	# -3.38 ± 1.39 –3.25 (-1 to -6) -2.72 to -4.03	# -0.48 ± 0.39 –0.5 (-1 to 0.5) -0.29 to -0.66
**12 m post-op** Mean ± SD Median (Range) 95% CI	# -5.02 ± 3.7 –4.25 (-13.25 to 0.5) -3.29 to -6.76	# -0.61 ± 0.44 –0.75 (-1.25 to 0.5) -0.41 to -0.82	# -3.24 ± 1.5 -3 (-1 to -6.5) -2.54 to -3.94	# -0.51 ± 0.41 –0.5 (-1 to 0.5) -0.32 to -0.7
**Δ spherical equivalent/ cylindrical refraction** Mean ± SD Median (Range) 95% CI	3.18 ± 2.41 2.75 (-0.5 to 7) 2.05 to 4.3	7.21 ± 6.13 7.13 (-12.75 to 17) 4.34 to 10.08	2.4 ± 1.65 2.5 (-1 to 5.5) 1.63 to 3.17	3.15 ± 1.47 2.88 (0.75 to 5.5) 2.46 to 3.84
**P value**	< 0.001*	< 0.001*	< 0.001*	< 0.001*

* significant difference between 12 months post-operative and baseline data at p value < 0.05

#: significant difference between each time point and baseline at p value < 0.05

**Table 2:** Results of uncorrected (UCVA) and best corrected (BCVA) visual acuity in both groups

	UCVA		BCVA	
	ICRS	TICL	ICRS	TICL
	N = 20	N = 20	N = 20	N = 20
**Baseline** Mean ± SD Median (Range) 95% CI	1.15 ± 0.29 1.3 (0.5 to 1.3) 1.01 to 1.28	1.19 ± 0.13 1.24 (1 to 1.3) 1.13 to 1.25	0.57 ± 0.22 0.5 (0.2 to 1) 0.47 to 0.67	0.33 ± 0.14 0.3 (0.2 to 0.6) 0.27 to 0.39
**1 m post-op** Mean ± SD Median (Range) 95% CI	# 0.9 ± 0.28 1 (0.4 to 1.3) 0.76 to 1.03	# 0.36 ± 0.18 0.35 (0.1 to 0.7) 0.27 to 0.44	# 0.34 ± 0.2 0.3 (0.1 to 1) 0.24 to 0.44	0.32 ± 0.17 0.3 (0.1 to 0.7) 0.24 to 0.4
**3 m post-op** Mean ± SD Median (Range) 95% CI	# 0.92 ± 0.25 1 (0.5 to 1.3) 0.8 to 1.03	# 0.34 ± 0.17 0.3 (0.1 to 0.7) 0.26 to 0.42	# 0.38 ± 0.22 0.3 (0.1 to 1) 0.27 to 0.48	0.32 ± 0.18 0.3 (0.1 to 0.7) 0.23 to 0.4
**6 m post-op** Mean ± SD Median (Range) 95% CI	# 0.9 ± 0.3 1 (0.4 to 1.3) 0.8 to 1	# 0.3 ± 0.1 0.3 (0.1 to 0.6) 0.2 to 0.4	# 0.34 ± 0.15 0.3 (0.1 to 0.8) 0.27 to 0.41	# 0.26 ± 0.14 0.2 (0.1 to 0.6) 0.2 to 0.32
**12 m post-op** Mean ± SD Median (Range) 95% CI	# 0.9 ± 0.26 1 (0.4 to 1.3) 0.77 to 1.02	# 0.31 ± 0.15 0.25 (0.1 to 0.6) 0.23 to 0.38	# 0.33 ± 0.2 0.3 (0.1 to 1) 0.24 to 0.42	# 0.27 ± 0.13 0.2 (0.1 to 0.6) 0.21 to 0.33
**Δ UCVA/BSCVA** Mean ± SD Median (Range) 95% CI	-0.25 ± 0.17 –0.3 (-0.6 to 0) -0.17 to -0.33	-0.88 ± 0.17 –0.84 (-0.58 to -1.2) -0.8 to -0.96	-0.24 ± 0.14 –0.25 (-0.5 to 0) -0.17 to -0.31	-0.06 ± 0.08 0 (-0.2 to 0) -0.02 to -0.1
**P value**	< 0.001*	0.001*	< 0.001*	0.006*

* significant difference between 12 months post-operative and baseline data at p value < 0.05

#: significant difference between each time point and baseline at p value < 0.05

**Fig. 1:**
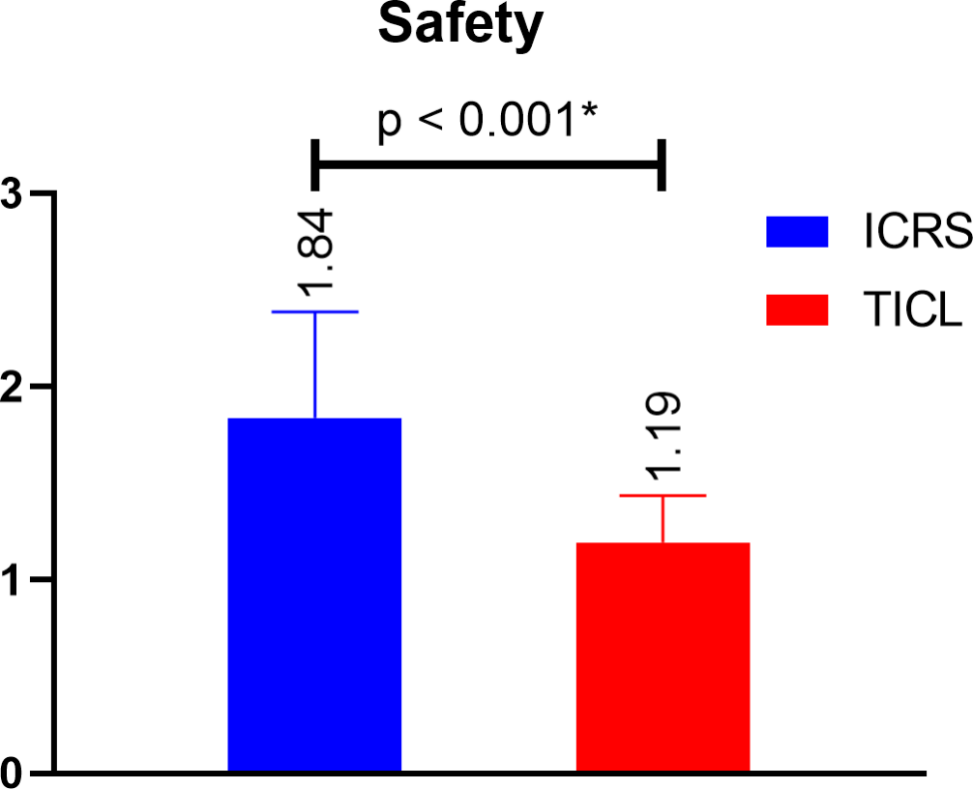
Comparison of safety outcomes between both study groups

**Fig. 2:**
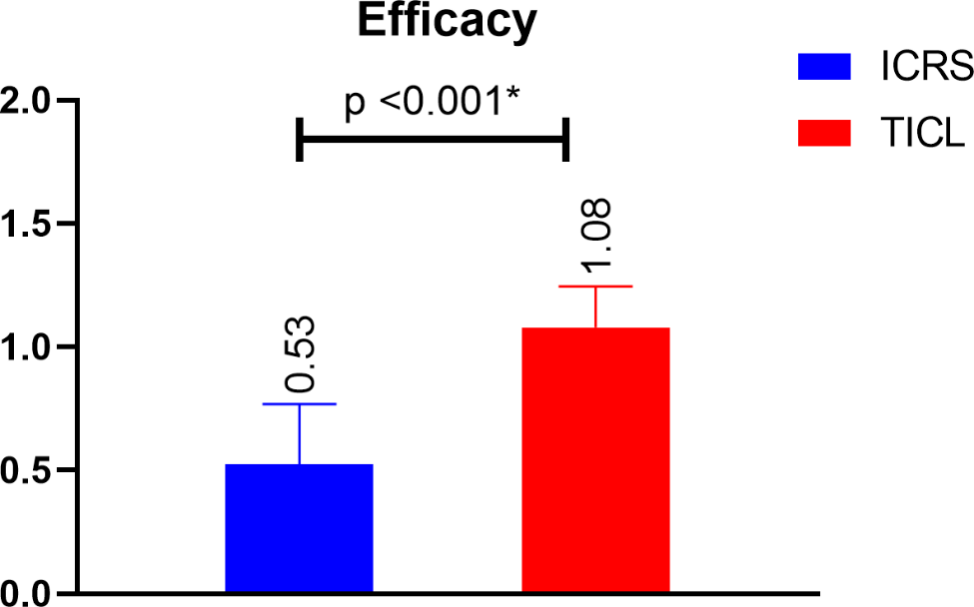
Comparison of efficacy outcomes between both study groups

We found high safety value in ICRS group (1.84 compared with 1.19) while in TICL group there were high efficacy values (1.08 compared with 0.53) and that was statistically significant in both comparisons as shown in Figs. 1 and 2.

## Discussion

In keratoconus cases, near-normal corneal biomechanics and surface regularity are very important factors for the expected postoperative visual acuity. Patients with highly abnormal corneas have poor BCVA and thus poor expected postoperative UCVA. Different treatment modalities in keratoconus aim to stabilize the case and get the highest possible visual acuity values, ICRS as one of these modalities act as an add tissue in the corneal deep stroma in the mid-periphery forming tension forces on the stromal collagen to regain the normal corneal shape and regularity as much as possible and it was successful as evidenced by different studies with improvement in spherical equivalent, cylindrical refraction and so in visual acuity [7, 11, 12].

In this study for the ICRS group, the mean improvements in spherical equivalent, cylindrical refraction, UCVA and BCVA were 3.18 D, 2.4 D, 0.25 (log MAR) and 0.24 (log MAR) respectively which is comparable and even slightly better than other studies [11, 12]. Prisant et al. [13] in 2019, reported an improvement (within 3 months) in spherical refraction, cylindrical refraction, UCVA and BCVA of 0.84D, 2.21D, 0.4 (log MAR) 0.1 (log MAR) respectively. In this study, BCVA showed a higher improvement which may be related to the stability and high values of the sphero-cylindical correction while regarding UCVA, our study showed less improvement of 0.25 log MAR which may be related to the preoperative high errors.

Regarding ICL implantation in keratoconic eyes, the refractive accuracy in such patients is less and refractive surprises are more than in normal non-keratoconic due to the high variability in the subjective refraction on which the TICL power and axis of insertion is based on [14].

In this study, efficacy index was 1.08 and safety index was 1.19 and so, our results are comparable to the published studies on the outcomes of ICL implantation in keratoconus [15–19]

Hashemian et al. [20] showed better efficacy and safety indices of 1.345 and 1.56 respectively at 1 year postoperative. These differences could be explained by the different preoperative data reported by Hashemian et al. which indicates milder cases because in our study the preoperative spherical equivalent and cylindrical ranges were from − 11.75 D to −17.75 D and from 1.00 D to 6.00 D respectively, while in that study the preoperative spherical equivalent and cylindrical ranges were from − 1.50D to −10.25 D and from 0.50 D to 6.5 D. Alfonso et al. implant ICL in mild keratoconus and had nice refractive outcomes with very satisfactory efficacy index (1.07) and safety index (1.16) which is comparable to our results as mentioned before [21]. Alio et al. demonstrated an efficacy index of 0.88 and safety index of 1.21 for patients who received an ICL for keratoconus [22]. Antonios et al. reported an overall efficacy index of 1.04 and a safety index of 0.72 after ICL implantation 2 years postoperative [18].

In 2018, Ramin et al. [23] compared visual and refractive including aberration measurements of ICRS versus TICL Lens Implantation in mild and moderate cases of keratoconus and their preoperative data for the ICRS group regarding UCVA (log-Mar), BCVA (log-Mar), spherical equivalent (D) and astigmatism (D) were 0.65, 0.37, −5.70 and 6.25 respectively and one year postoperative data for these measurements were 0.39, 0.35, −3.67 and 4.1 respectively compared with the TICL group with preoperative data for the same measurements respectively 0.82, 0.15, −8.57 and 4.83 and one year postoperative data were 0.13, 0.04, −0.97 and 0.97 respectively. With comparison of these data with this study, lower preoperative UCVA and BCVA of 1.15 and 0.57 for ICRS group were noted compared with 1.19 and 0.33 for TICL group respectively which is explained in ICRS group by the preoperative mean spherical equivalent which was higher (−8.2) and as usual keratoconic eyes data are unexpected specially preoperative due to presence of another factors that is not totally related to the refraction which are the aberrations. Our postoperative data (12 months) regarding logMar UCVA, logMar BCVA, spherical equivalent (D) and astigmatism (D) for ICRS group were 0.9, 0.33, −5.02 and 3.24 respectively and for TICL group were 0.31, 0.27, −0.61 and 0.51. These differences in visual acuity (preoperative and postoperative) may be due to the better preoperative case data as that mild keratoconic changes contributes in less aberrations and better postoperative results. Both studies in general prove the significant superiority of TICL over ICRS in the UCVA, which was expected by the expected spherical equivalent correction but the main critical difference was that in this study there was a higher improvement in the BCVA in the Kera-ring group and the BCVA which is the main treatment goal of cases of keratoconus which may be explained by the difference in modality nature because Kera-rings remodel (flatten) the corneal surface and so, correct some of the refractive error and decrease aberrations and so get more improvement differences in BSCVA.

Limitations of this study were its relatively short follow up period and low number of patients. Also, for future studies, it is better to use more comprehensive examinations, such as assessment of high ordered aberrations to obtain better results.

## Data Availability

I don’t wish to share my data and any reviewer wants to contact me and discuss my raw data, please kindly contact me on drahmedmaher1988@gmail.com.
